# Ethnic inequities in 6–8 week baby check coverage in England 2006– 2021: a cohort study using the Clinical Practice Research Datalink

**DOI:** 10.3399/BJGP.2023.0593

**Published:** 2024-07-23

**Authors:** Claire X Zhang, Maria A Quigley, Clare Bankhead, Chun Hei Kwok, Nikesh Parekh, Claire Carson

**Affiliations:** NIHR Policy Research Unit in Maternal and Neonatal Health and Care, National Perinatal Epidemiology Unit, Nuffield Department of Population Health, University of Oxford, Oxford.; NIHR Policy Research Unit in Maternal and Neonatal Health and Care, National Perinatal Epidemiology Unit, Nuffield Department of Population Health, University of Oxford, Oxford.; Nuffield Department of Primary Care Health Sciences, University of Oxford, Oxford.; University of Oxford, Oxford, and Applied Health Research Unit, Nuffield Department of Population Health, University of Oxford, Oxford.; Royal Borough of Greenwich, London.; NIHR Policy Research Unit in Maternal and Neonatal Health and Care, National Perinatal Epidemiology Unit, Nuffield Department of Population Health, University of Oxford, Oxford.

**Keywords:** 6–8 week baby check, 6–8 week maternal postpartum check, ethnicity, preventive medicine, primary health care, vaccination

## Abstract

**Background:**

Inequities in the coverage of 6–8-week maternal checks, health visitor reviews, and infant vaccinations have been reported in England. Ethnic inequities in 6–8-week baby checks have not been studied nationally.

**Aim:**

To examine the effect of maternal ethnicity on 6–8 week baby check coverage in England 2006–2021.

**Design and setting:**

This cohort study used electronic health records from primary care in England.

**Method:**

Baby check coverage was calculated in 16 ethnic groups, by year and region. Risk ratios were estimated using modified Poisson regression. Coverage and timing of baby checks in relation to maternal checks and infant vaccinations by ethnic group were calculated.

**Results:**

Ethnic inequities in 6–8 week baby check coverage in England varied by year and region. Coverage increased 2006–2007 to 2015–2016, then stabilised to 80–90% for most groups. Coverage was lowest for Bangladeshi and Pakistani groups 2006–2007 to 2011–2012. In the West Midlands, coverage was lowest at approximately 60% for four groups: Bangladeshi, Caribbean, African, and Any other Black, African or Caribbean background. In the North West, coverage was lowest for Bangladeshi (65.3%) and Pakistani (69.2%) groups. These patterns remained after adjusting for other factors and persisted over time. Coverage was highest in those whose mothers received a maternal check and those who received at least one dose of 8-week infant vaccinations.

**Conclusion:**

Coordinated action at the level of integrated commissioning boards, primary care networks, and GP practices is required to better understand the reasons behind these inequities and redress the persistent disparities in 6–8 week baby check coverage.

## Introduction

Various preventive care services are provided in England for mothers and babies 6–8 weeks after birth. These include the 6–8 week baby check for physical examination,^1^ the 6–8 week maternal check for postnatal physical and mental health concerns,^2^ the 6–8 week health visitor review for breastfeeding, sleep, and support at home,^3^ and the first infant vaccinations at 8 weeks.

GP practices, health visiting services, midwifery, and other community health services aim to provide coordinated care for mothers and babies. However, in practice, there are local variations in commissioning and delivery as well as changes to funding and clinical guidance over time.^2^^,^^4^ As a result, gaps and overlaps have been reported, leading to confusion for both service providers and parents during the postnatal period.^5^^,^^6^

Evidence of inequities in coverage by ethnicity have also emerged. Routine statistics showed that the odds of receiving a 6–8 week health visitor review was highest for ‘White’ infants compared with all other aggregated ethnic groups in 2018/2019 and 2019/2020, and the reverse was true for ‘Black’ infants.^7^ This was consistent across most regions. Lack of, and delays in, coverage of 6–8 week maternal checks are also patterned by ethnicity, deprivation, maternal age, and preterm birth.^8^^,^^9^ Additionally, stark ethnic inequities have been identified in childhood vaccinations across England.^10^^,^^11^ Although a study in London found preliminary evidence for ethnic inequities in 6–8 week baby checks during the first year of the COVID-19 pandemic,^12^ ethnic inequities in baby checks provided by GPs are yet to be examined at a national level.

This study investigated the effect of maternal ethnicity on infants receiving their 6–8 week baby checks at a GP practice in England between 2006 and 2021. Mother–baby linked electronic health records (EHRs) from GP and hospital data were used to estimate the percentage of infants born to mothers in each ethnic group receiving a 6–8-week baby check and how this varied by year and region. A secondary analysis described the relationship between coverage and timing of 6–8 week baby checks, 6–8 week maternal checks, and 8-week infant vaccinations by ethnic group.

**Table:** How this fits in

A number of preventive care services for infants and mothers are scheduled in the 6–8 week period after birth. Although ethnic inequities have been reported in the coverage of 6–8 week maternal checks, health visitor reviews, and infant vaccinations in England, little is known about whether 6–8 week baby checks are similarly affected. This study examined the relationship between ethnicity and 6–8 week baby checks, using electronic health record data from 2006 to 2021. The study found that ethnic inequities varied by year and region, with persistent inequities particularly affecting the Bangladeshi and Caribbean groups in the West Midlands and the Bangladeshi group in the North West in recent years. Across all ethnic groups, baby check coverage was highest for those who also received their 8-week vaccinations and whose mothers received a 6–8 week maternal check.

## Method

### Study design

This observational cohort study used individual-level data spanning the financial years 2006–2007 through 2020–2021.

### Data sources

In this study, data from the Clinical Practice Research Datalink (CPRD) Aurum primary care database, May 2022, was used; this included the CPRD Mother–Baby Link to link mother and baby records and the CPRD Pregnancy Register to obtain pregnancy and birth-related data. Ethnicity was derived from CPRD data linked to Hospital Episode Statistics (HES) Admitted Patient Care (APC) data. Other sociodemographic variables were obtained from linked Office for National Statistics (ONS) Index of Multiple Deprivation and Rural–Urban Classification data.

### Study cohort

**Figure 1.**
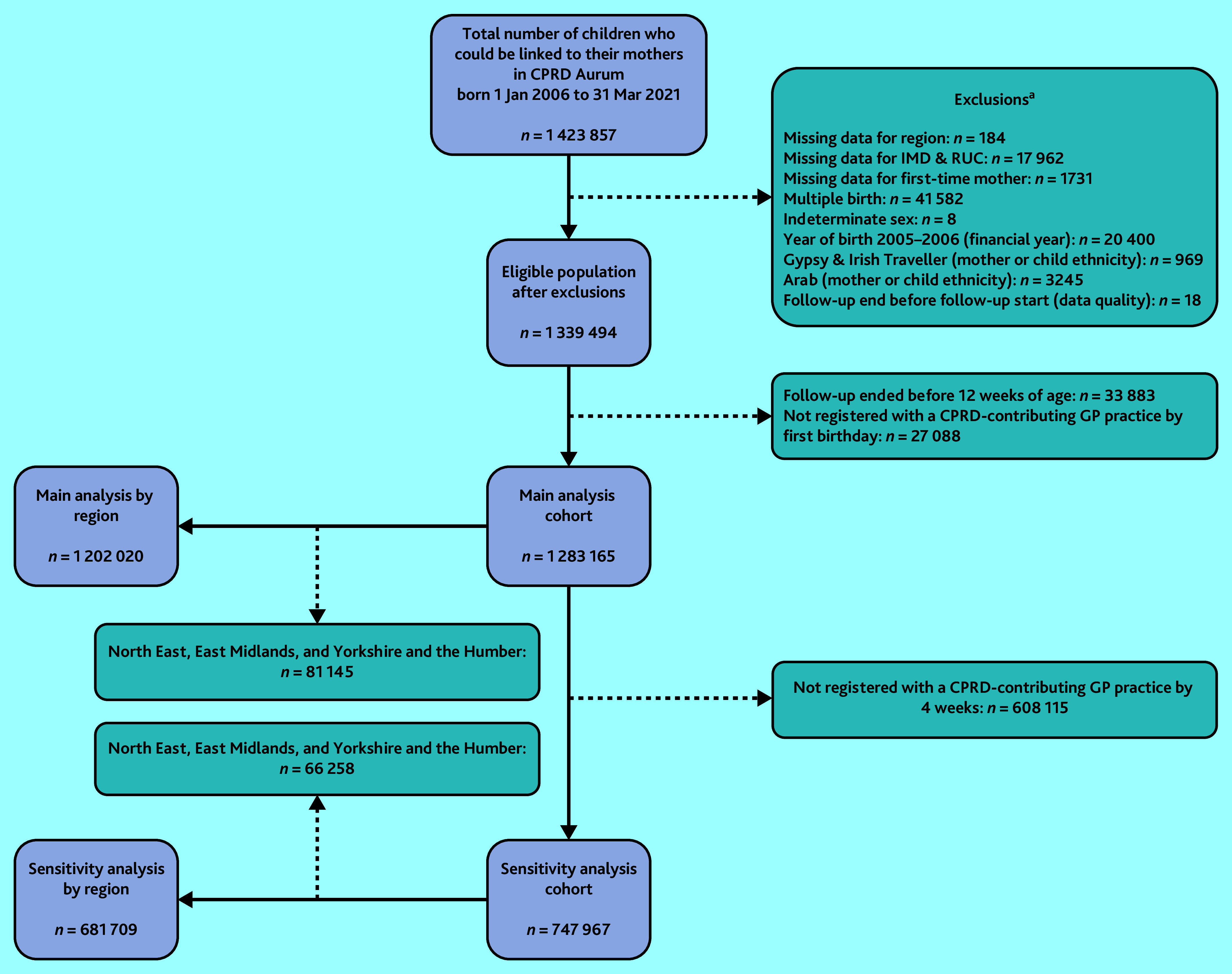
Study cohort inclusions and exclusions. ^a^Number of children excluded are not mutually exclusive. CPRD = Clinical Practice Research Datalink. IMD = Index of Multiple Deprivation. RUC = Rural–Urban Classification.

Children born after 1 January 2006 linked to their mothers using the CPRD Aurum Mother–Baby Link were the population eligible for this study. Follow-up began at birth and ended at the earliest of: de-registration from their GP practice, end of study period (31 March 2021), or death. Baby checks occurring at any time between 4 and 12 weeks after birth were identified to allow some leeway for the CPRD’s estimation of birth dates. Hence, the study excluded children whose follow-up ended before 12 weeks of age. For the main analysis, the study also restricted the eligible population to only those who were registered by 12 months of age (Figure 1). Some additional exclusions were applied because of small numbers in categorical variables or missing data. Before applying these exclusions, those with missing and non-missing data were compared by ethnic group and study outcomes in order to explore whether data were missing completely at random.

### Deriving study variables

Using code lists, 6–8 week baby check and maternal check codes were identified, expanding on previously published sources.^8^^,^^13^^,^^14^ Multiple codes recorded on the same date were de-duplicated. Further data cleaning is detailed in Supplementary Box S1.

Maternal ethnicity was chosen as the explanatory factor of interest since maternal/parental ethnicity has a greater bearing on preventive care for babies than babies’ ethnicity.^15^ First, ethnicity records in the CPRD were identified using an adapted code list from previously published sources.^16^^–^^18^ Where individuals had missing records or ethnicity coded as ‘unknown’ or ‘not stated’ in the CPRD, their HES APC episode-level ethnicity data were used. Where individuals had multiple ethnicity records, an algorithm to identify a single most plausible ethnic category was used.^19^^,^^20^ The ONS England and Wales Census disaggregated ethnicity classification system was used, with the exception of the Gypsy and Irish Traveller, Arab, and Roma ethnic groups as group sizes were too small for analyses and the representativeness of these ethnic groups in EHRs was a concern (see Supplementary Box S1 for further information). Sixteen ethnic groups and an ‘Unknown’ group were therefore included.

**Figure 2.**
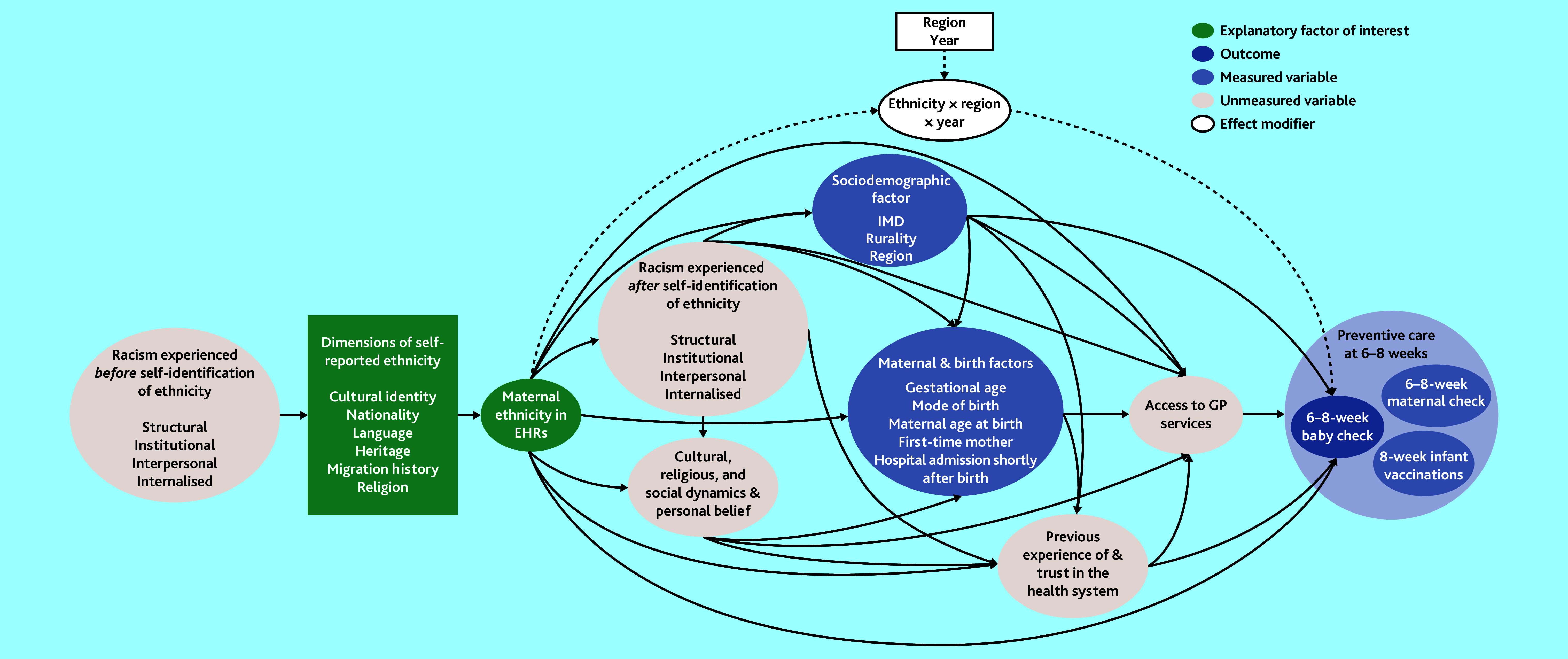
Directed acyclic graph for the effect of maternal ethnicity on 6–8 week baby checks in England. EHR = electronic health record. IMD = Index of Multiple Deprivation.

Other study variables derived from the linked dataset are detailed in Figure 2 and Supplementary Box S1.

### Statistical analysis

Coverage by ethnic group (the percentage of children born to mothers in each ethnic group who received a 6–8 week baby check) overall, stratified by year, and stratified by region were calculated. If a region contained ethnic group(s) with less than five children who had not received a baby check, this entire region was excluded from analyses involving regional stratification. Coverage was then stratified by both year and region to describe trends over time. Ethnic groups within each region and year that contained less than five children who had not received a baby check were excluded from this analysis.

Using risk ratios (RRs) from modified Poisson modelling with robust standard errors, the average (total) effect of maternal ethnicity on receiving a baby check was calculated. As there was effect modification by region (likelihood ratio test *P*<0.001), RRs stratified by region are reported, comparing each ethnic group to the English, Welsh, Scottish, Northern Irish, or British group (hereon referred to as the White British group) in that same region. Although exploration of effect modification by both region and year was initially planned, ethnic group sizes were too small to proceed.

A directed acyclic graph was constructed informed by the existing evidence to guide statistical modelling (Figure 2).^21^^–^^29^ Two models were run: one without variable adjustment, and one with multivariable adjustment for all measured sociodemographic and maternal/birth-related factors.

To address the study’s secondary research question, the percentage of children in each ethnic group that received a baby check was described, stratified by whether their mothers received a maternal check, and whether they received at least one dose of an 8-week infant vaccination. The proportion of children who received their baby check on the same day as an infant vaccination and/or their mother’s maternal check was calculated.

Sensitivity analyses were conducted by replicating the above for a cohort of children registered by 4 weeks of age to determine whether delayed retrospective coding of 6–8 week baby checks by GP practices affected the findings.

Further methodological detail is included in Supplementary Box S1 and published in a related study.^11^

## Results

### Cohort characteristics

Of the 1 339 494 eligible children, 1 283 165 (95.8%) were included in the main analysis and 747 967 (55.8%) in sensitivity analysis. Maternal ethnicity was largely representative of the general population of women of child-bearing age (15–49 years) and infants at the time of the 2011 Census (Table 1),^30^ as were most other sociodemographic, maternal, and birth factors (Supplementary Table S1). There was a slightly lower proportion of children born preterm in the current study cohort. North East, East Midlands, and Yorkshire and the Humber were excluded from analyses involving regional stratification because of small group sizes. There were no notable differences in patterns of missing data between ethnic groups or between those who did and did not receive baby checks.

**Table 1.** Distribution of ethnicity in the study cohort compared with the general population of England^a^

**Maternal ethnicity**	**Reference population,^b^ %**	**Main analysis, *n* (%)^c^ (*N* = 1 283 165)**	**Sensitivity analysis, *n* (%)^d^ (*N* = 747 967)**
**English, Welsh, Scottish, Northern Irish, or British**	74.8	877 583 (68.4)	525 349 (70.2)
**Irish**	0.8	6757 (0.5)	3784 (0.5)
**Any other White background**	6.9	118 485 (9.2)	69 537 (9.3)
**Indian**	3.1	41 418 (3.2)	22 726 (3.0)
**Pakistani**	2.4	39 020 (3.0)	21 351 (2.9)
**Bangladeshi**	0.9	17 411 (1.4)	10 359 (1.4)
**Chinese**	1.1	9904 (0.8)	5202 (0.7)
**Any other Asian background**	2.1	31 833 (2.5)	17 364 (2.3)
**Caribbean**	1.3	12 964 (1.0)	5904 (0.8)
**African**	2.5	53 582 (4.2)	26 391 (3.5)
**Any other Black, African or Caribbean background**	0.6	8410 (0.7)	4074 (0.5)
**White and Black Caribbean**	0.8	8433 (0.7)	4237 (0.6)
**White and Black African**	0.3	4952 (0.4)	2505 (0.3)
**White and Asian**	0.6	4171 (0.3)	2337 (0.3)
**Any other Mixed or multiple ethnic background**	0.6	8493 (0.7)	4503 (0.6)
**Any other ethnic group**	0.7	25 860 (2.0)	14 110 (1.9)
**Unknown**	N/A	13 889 (1.1)	8234 (1.1)

a *Denominator is number of children.*

b *Reference population: women aged 15–49 years in England at the time of the 2011 Census.*

c *Main analysis includes those registered with a CPRD GP practice by 12 months of age.*

d *Sensitivity analysis includes those registered with a CPRD GP practice by 4 weeks of age. CPRD = Clinical Practice Research Datalink. N/A = not applicable.*

### Six-to-eight-week baby checks by maternal ethnicity, year, and region

The effect of ethnicity varied over time and by region. Coverage increased between 2006–2007 and 2015–2016, then stabilised between approximately 80% and 90% across the rest of the study period for most ethnic groups (Supplementary Figure S1 and Supplementary Table S2).

However, between 2006–2007 and 2010–2011, a much lower proportion of children born to mothers of Bangladeshi (for example, 52.7% compared with 68.7% in the White British group in 2006–2007) and Pakistani (for example, 57.0% in 2006–2007) ethnicity received the baby check. This gap between Pakistani and Bangladeshi groups and other ethnic groups was even greater in the sensitivity analysis (Supplementary Figure S2 and Supplementary Table S3).

**Figure 3.**
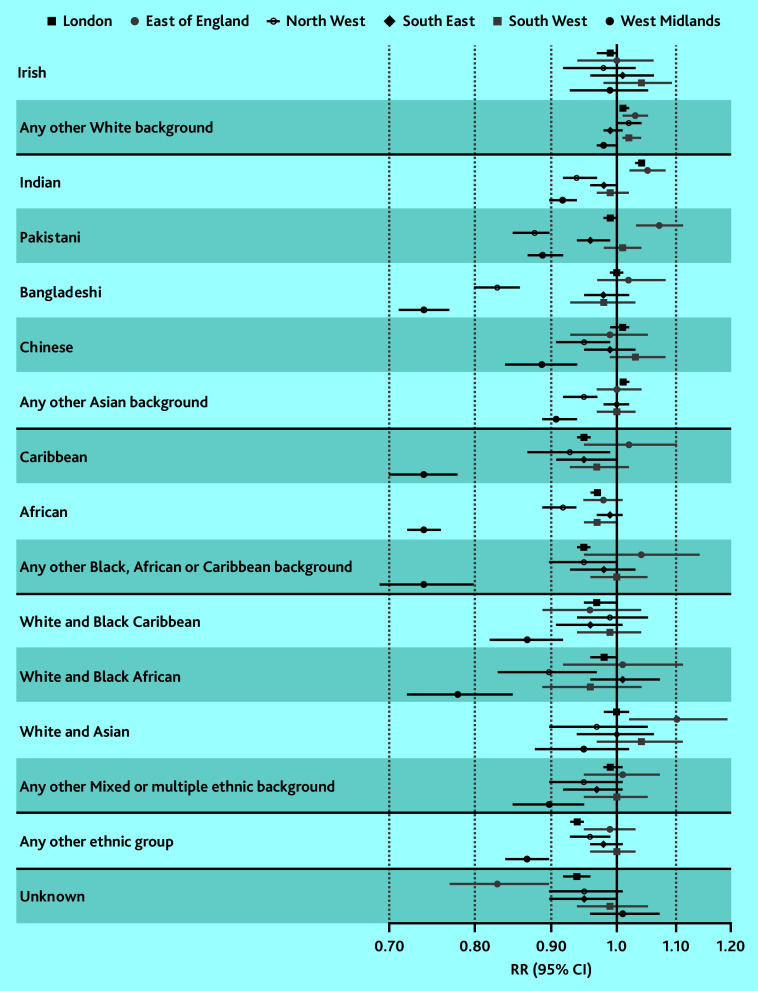
Average (total) effect of maternal ethnicity on 6–8 week baby checks, accounting for effect modification by region (comparing each ethnic group with the White British reference group in the same region). CI = confidence interval. RR = risk ratio.

By region, lower coverage and greater disparities between ethnic groups were observed in the West Midlands and the North West (Supplementary Figure S3 and Supplementary Table S4). In the West Midlands, baby check coverage was the lowest for four ethnic groups where only 59.3–60.2% of babies received the check: Bangladeshi, Caribbean, African, and Any other Black, African or Caribbean background (RR 0.74, 95% confidence intervals [CIs] ranging from 0.7 to 0.8 compared with White British; Figure 3, Supplementary Figure S3, and Supplementary Tables S4 and S5). In contrast, coverage was 80.5% in the White British group.

In the North West, baby check coverage was lowest for the Bangladeshi (65.3%, RR 0.83, 95% CI = 0.8 to 0.86 compared with White British) and Pakistani ethnic groups (69.2%, RR 0.88, 95% CI = 0.85 to 0.9). Coverage was 79.0% in the White British group. These patterns were similar in sensitivity analysis (Supplementary Figures S2, S4, and S5 and Supplementary Tables S3, S6, and S7) and remained consistent after adjusting for sociodemographic, maternal, and birth factors (Supplementary Figures S6 and S7 and Supplementary Tables S8 and S9).

**Figure 4.**
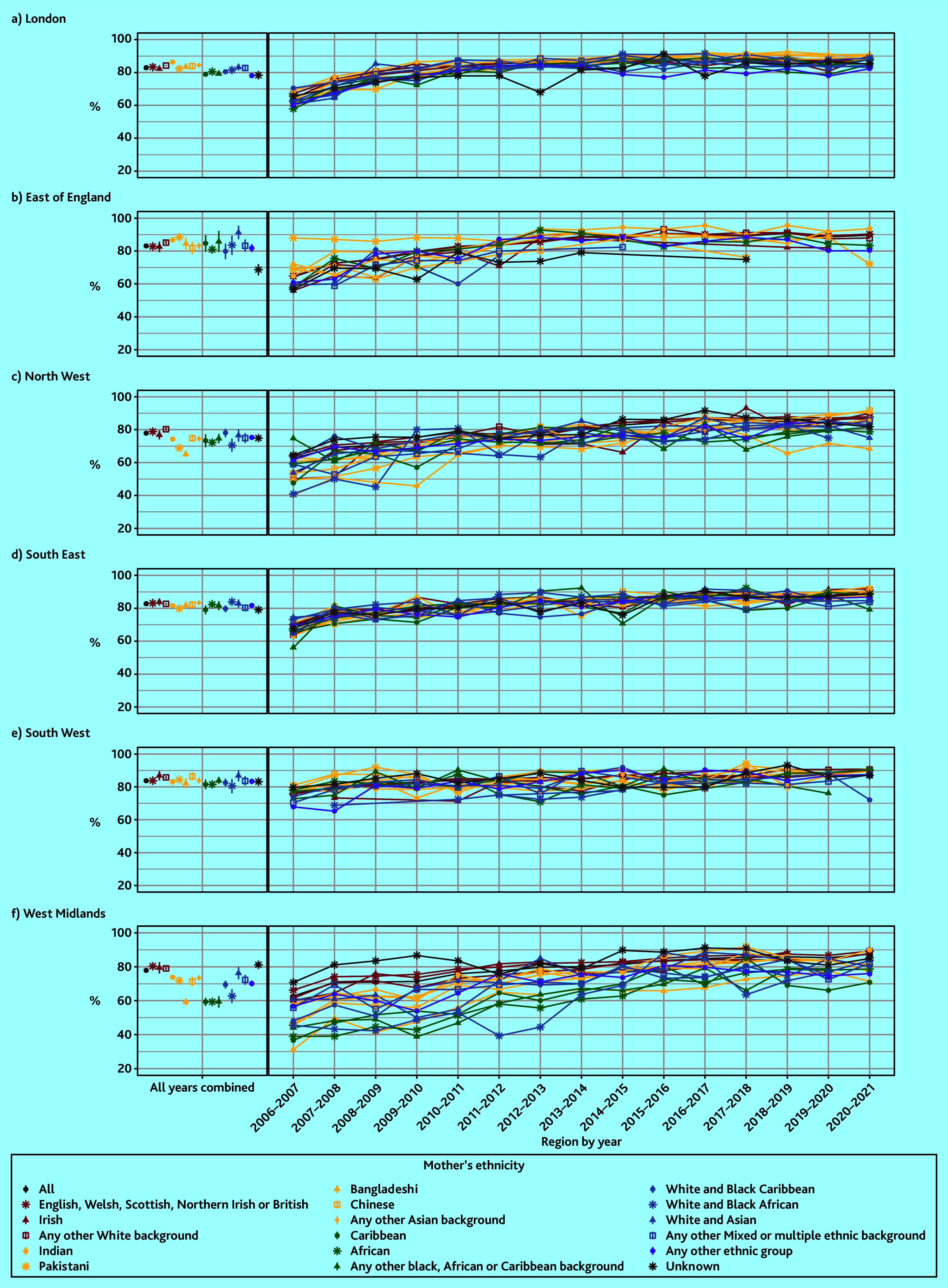
Six-to-eight-week baby check by maternal ethnic group, region, and year. Data points for ethnic groups within each region and year with less than five children who had not received a baby check have been removed. a) London; b) East of England; c) North West; d) South East; e) South West; f) West Midlands.

In the West Midlands and North West, ethnic inequities persisted over time (Figure 4, Supplementary Table S10). For example, the Bangladeshi and Caribbean groups still had lower coverage than White British in the West Midlands in 2020–2021 (71.2% coverage, 95% CI = 62.4 to 79.9% for Bangladeshi and 70.8%, 95% CI = 58 to 83.7% for Caribbean groups compared with 89.3%, 95% CI = 88.4 to 90.1% for the White British group). In the North West, coverage in the Bangladeshi group improved in the mid-2010s then declined again in recent years (for example, 68.4% coverage, 95% CI = 60.5 to 76.3% in 2020–2021 compared with 87.8%, 95% CI = 87 to 88.6% in the White British group).

### Six-to-eight-week baby checks, maternal checks, and infant vaccination

Six-to-eight-week maternal check coverage was lower than baby checks, between 58.0% and 68.0% across ethnic groups. Pakistani (58.0%) and Any other Black, African or Caribbean background (60.4%) had the lowest maternal check coverage (Supplementary Tables S11 and S12).

**Figure 5.**
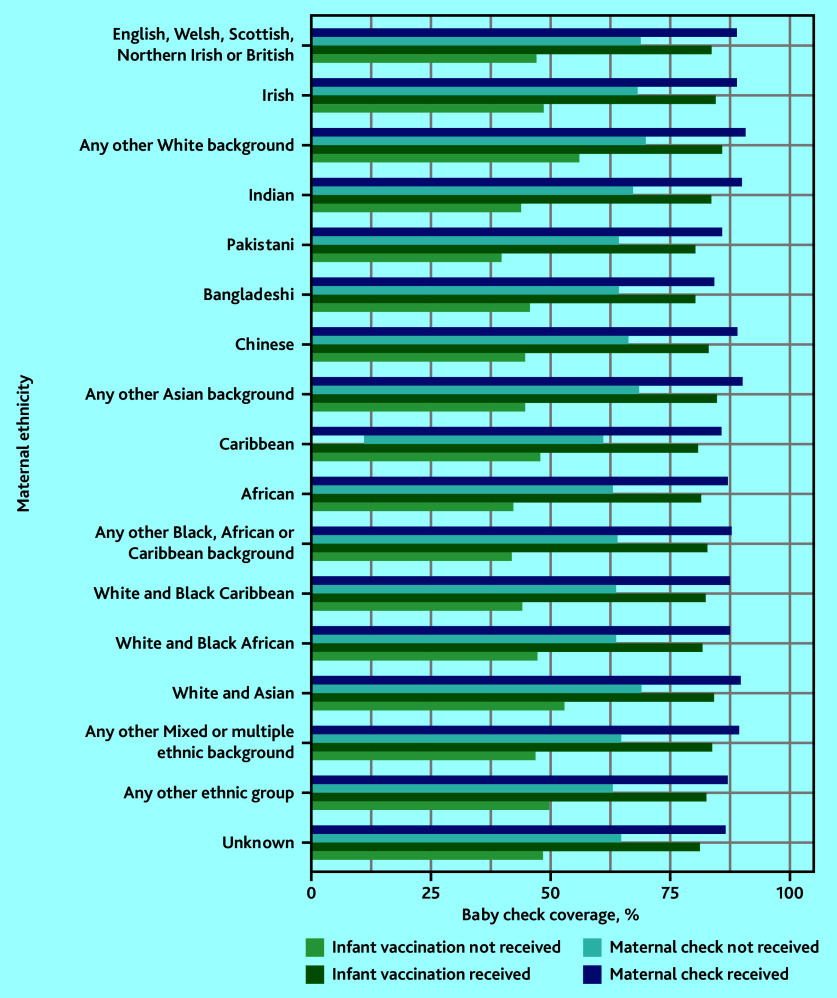
Six-to-eight-week baby check coverage by ethnic group, stratified by receipt of maternal checks and infant vaccination.

Baby check coverage was lower for those whose mothers did not receive a maternal check, particularly for the Caribbean group. Baby check coverage was also lower in those who did not receive at least one dose of an infant vaccination, particularly for the Pakistani group (Figure 5, Supplementary Figure S8). Across all ethnic groups, the gap in baby check coverage was wider between children who did and did not receive infant vaccination than between children whose mothers did and did not receive maternal checks.

Of babies receiving a 6–8 week check, approximately three-quarters received at least one other service on the same day (Supplementary Figures S9 and S10). This was consistent across ethnic groups, with slight differences in the proportion of each ethnic group receiving different combinations of services on the same day. For example, the Pakistani group had the lowest proportion of babies and mothers receiving their 6–8 week checks on the same day (23.6% compared with 35.1% in the White British group). Bangladeshi and Any other White background had the lowest proportion of babies receiving their baby check on the same day as infant vaccination (24.8%), although this was similar to that of the White British group (26.2%)

## Discussion

### Summary

Ethnic inequities in 6–8 week baby check coverage in England has varied across time and by region. Overall, a lower proportion of babies born to mothers of Bangladeshi and Pakistani ethnicity received a 6–8 week baby check than other ethnic groups between 2006–2007 and 2010–2011 (for example, 52.7% and 57.0% compared with 68.7% in the White British group in 2006–2007). In the West Midlands, coverage was lowest for four ethnic groups: Bangladeshi, Caribbean, African, and Any other Black, African or Caribbean background (59.3–60.2% compared with 80.5% for White British). In the North West, this was the case for Bangladeshi and Pakistani ethnic groups (65.3% and 69.2% compared with 79.0% for White British). These patterns remained consistent after adjusting for sociodemographic factors and maternal and birth factors, and persisted over time, particularly for the Bangladeshi and Caribbean groups in the West Midlands and the Bangladeshi group in the North West.

Three-quarters of babies who received a 6–8 week check also received an infant vaccination and/or maternal check (for their mother) on the same day. Babies in all ethnic groups were less likely to receive a check if their mothers did not have a maternal check, or if they did not receive at least one dose of an infant vaccination. This affected some ethnic groups slightly more than others.

### Strengths and limitations

This is the first study, to the authors’ knowledge, to use EHRs to examine ethnic inequities in 6–8 week baby check coverage across England. Given the large size of the CPRD Aurum and linkage of mother and baby records, it was possible to examine maternal ethnicity at a disaggregated level to provide more granular findings than previous studies.^31^ Examining maternal ethnicity also has greater potential to inform public health action as parental ethnicity has a greater bearing on infants’ preventive care utilisation than the ethnicity of the infant.^15^

Baby check coverage is not monitored nationally, so it is difficult to determine whether overall trends (for example, increase in prevalence) are an artefact of improved clinical coding or improved coverage. Future studies of 6–8 week checks should also consider study designs that better represent regions like North East, East Midlands, and Yorkshire and the Humber, as well as Arab, Roma, and Gypsy and Irish Traveller ethnic groups.

### Comparison with existing literature

The scarce existing evidence on 6–8-week preventive care services used aggregated ethnic groups, so it is difficult to make comparisons with the current study. Babies from ‘Black’ ethnic groups are least likely to receive health visitor checks,^7^ and, during the pandemic, lower baby check coverage was found for the ‘Black Other’ group compared with ‘White British/Irish’ in London;^12^ this was also the case in the current study for baby checks in the West Midlands.

### Implications for research and practice

Although the inclusion of 6–8 week baby checks and maternal checks as essential services in the NHS England General Medical Services contract in 2020/2021 is a welcome change to boost coverage overall,^4^^,^^32^^,^^33^ funding for GPs alone is unlikely to redress the persistent ethnic inequities in coverage. Given regional disparities and the lack of evidence on why some ethnic groups are more affected by inequities in preventive care checks, localised action is required to better understand and redress the modifiable factors that drive these inequities.

For example, there may be a lack of clarity for parents on the purpose of each of the various preventive care services during this 6–8 week period,^5^^,^^6^ particularly when information about services may have been provided in ways that were not responsive to language, cultural, religious, and gender preferences, health literacy levels, and digital access.^15^ Providing such information during this stressful early infancy period can also make it challenging for parents to make informed decisions,^11^ and may lead to parents seeking information and reassurance outside of the NHS. These factors could have been compounded by GP access barriers and mistrust of health services affecting some ethnic groups more than others.^34^ This includes factors that affect trust and decision making from pregnancy through to early childhood, such as the increased risk of infant mortality in babies born to Caribbean, African, Pakistani, and Bangladeshi mothers that has been documented nationally and specifically in the West Midlands.^35^ Better understanding of these complex, interwoven, and context-specific factors will help to ensure co-production of effective strategies to boost coverage for specific minority ethnic communities, including the tailoring of programmes that have been successfully implemented in other services and settings, such as providing information through trusted community leaders and co-locating services in community spaces.^36^

Additionally, given findings that baby check coverage is higher in those who received another preventive care service, local health services need a better understanding of whether and why different models of service delivery (for example, joint appointments, longer appointments) could act as a facilitator or barrier for preventive care access in some ethnic groups. The effectiveness of the disparate commissioning and service delivery arrangements across the country for these preventive care checks could be evaluated. Integrated commissioning boards and primary care networks could support GP, health visitor, and midwifery services to review how the various perinatal services are offered to families in their locality, whether the purpose of these appointments are clearly communicated for families from diverse language, cultural, and religious backgrounds,^15^ whether gaps still remain in meeting infants’ health needs and addressing parents’ concerns at the 6–8-week time point, and what can be done within their specific contexts to ensure equity of access and coverage.

Finally, the paucity of evidence and lack of comparability between studies and routine statistical reports calls for a unified approach to using ethnicity classification systems in research and monitoring, as well as standardised methods for coding preventive care checks in primary care.

## Data

All data used in this study were provided by the CPRD and cannot be shared with other researchers. Researchers must seek approval from CPRD to access this data for the purposes of their own research. This study is based in part on data from the CPRD obtained under licence from the UK Medicines and Healthcare products Regulatory Agency. The data were provided by patients and collected by the NHS as part of their care and support. Linked data were also provided by the Office for National Statistics (ONS). ONS and Hospital Episode Statistics data © (2021) were re-used with the permission of the Health & Social Care Information Centre. All rights reserved. The interpretation and conclusions contained in this study are those of the authors alone.
